# The Effects of Melatonin Administration on Intestinal Injury Caused by Abdominal Irradiation from Mice

**DOI:** 10.3390/ijms22189715

**Published:** 2021-09-08

**Authors:** Qin Wang, Yan Wang, Liqing Du, Chang Xu, Qiang Liu, Saijun Fan

**Affiliations:** Tianjin Key Laboratory of Radiation Medicine and Molecular Nuclear Medicine, Institute of Radiation Medicine, Chinese Academy of Medical Science, Tianjin 300192, China; wangqin@irm-cams.ac.cn (Q.W.); wangyan@irm-cams.ac.cn (Y.W.); dlq@irm-cams.ac.cn (L.D.); xuchang@irm-cams.ac.cn (C.X.); liuqiang@irm-cams.ac.cn (Q.L.)

**Keywords:** melatonin, intestinal injury, abdominal irradiation, radioprotector

## Abstract

Intestinal injury caused by ionizing radiation (IR) is a main clinical issue for patients with cancer receiving abdominal or pelvic radiotherapy. Melatonin (*N*-acetyl-5-methoxytryptamine) is a neurohormone that the pineal gland in the brain normally secretes. The study aimed to disclose the potential function of melatonin in intestinal injury induced by IR and its mechanism. Pretreatment with melatonin enhanced the 30-day survival rate of the irradiated mice and promoted the recovery of the intestinal epithelium and hematopoietic function following abdominal irradiation (ABI). Melatonin altered the gene profile of the small intestines from mice following ABI. The enriched biological process terms for melatonin treatment prior to radiation were mainly involved in the immune process. LPS/IL-1-mediated inhibition of RXR Function, TWEAK signaling, and Toll-like receptor signaling were the most activated canonical pathways targeted by melatonin. An upstream analysis network showed that Tripartite motif-containing 24 (TRIM24) was the most significantly inhibited and S100 calcium binding protein A9 (S100A9) activated. TRIM24 activated atherogenesis and cell viability in breast cancer cell lines and S100A9 inhibited the metabolism of amino acids. Melatonin has radioprotective effects on ABI-caused intestinal injury. The mechanisms behind the beneficial effects of melatonin were involved in activation of the immunity. It is necessary to conduct further experiments to explore the underlying mechanisms.

## 1. Introduction

Radiotherapy is a conventional and effective treatment for neoplasm in a clinical context. More than 50 percent of tumor patients receive radiotherapy for curative or palliative purposes [1]. During the radiotherapy of patients with abdominal or pelvic tumors, the small and large bowel is one of the crucial organs at risk during radiotherapy delivered to the abdomen and/or pelvis. A course of clinical radiotherapy usually lasts 4–6 weeks and the average exposure dose is about 50 Gy. As high dose of ionizing radiation (IR) can kill intestinal epithelial cells, intestinal absorption and barrier function may be destroyed and result in acute intestinal injury [2]. The major clinical manifestations of intestinal injury include abdominal pain, diarrhea, and water electrolyte imbalance. In severe cases, intestinal fistula, intestinal stenosis, intestinal obstruction, may occur and even be life-threatening. Thus, IR-caused intestinal injury limits the effective radiation dosage used to eradicate tumors and adversely affects the curative effect. 

Symptomatic treatment is commonly used to alleviate intestinal injury clinically. Actually, the main effective method to avoid bowel injury is to avoid intestine irradiation with better radiotherapy techniques (IMRT, IGRT, online adaptive RT). Radioprotective action such as use of radioprotectors, radiation mitigators, or therapeutic agents is usually undertaken during radiotherapy. A radioprotector as the earliest radiation countermeasure taken is usually given before radiation; thus, it is far superior to other agents in terms of therapeutic effect. Many natural and synthetic radioprotective agents have been applied for years, including ammonia and sulfur compounds, polyphenols, superoxide dismutase, cytokine, Chinese herbal medicine, etc. [3]. However, there are still some problems need to be solved, such as toxicity, stability, effectivity, and administration route. The best known radioprotector, amifostine, a kind of thiol compound, is the only clinically approved synthetic radioprotector [4]. However, amifostine may cause some side effects, including hypotension, nausea, vomiting, hot flashes, mild somnolence, and hypocalcemia, which limit its utility in clinical radiotherapy. To date, an effective and convenient radioprotective agent available to prevent or reduce radiation damage on the body has not been developed yet. It is necessary to explore an optimal radioprotector for individuals suffering from radiotherapy for tumors. 

Melatonin (*N*-acetyl-5-methoxytryptamine) is a neurohormone secreted by the pineal gland in the brain of mammals and humans. It was originally discovered to participate in the control of circadian rhythms and possibly sleep processes in diurnal species [5]. Thereafter, melatonin was reported to be involved in the regulation of lots of biological processes, including anti-aging, antioxidation, scavenging free radical, regulation of the immune system, and prevention of tumors [6]. Recent studies have indicated its effectiveness in resisting radiation in both in vitro and in vivo experiments [7]. Melatonin can not only safely be self-administered orally, sublingually, or via an intranasal spray, but can also be used in the short term or long term with little to no significant side effects. It is worth noting that melatonin was successfully used on radiation accident victims and workers in the radiation leak from the Fukushima Daiichi Nuclear Power Plant. Therefore, Das et al. inferred that melatonin may be a desirable candidate as a countermeasure against IR exposure [8]. 

To date, there have been a lot of reports about the potential of melatonin to protect against radiation. However, the literature on radiation-caused intestinal injury is limited. In this study, the effects of melatonin on intestinal injury caused by abdominal irradiation (ABI) from mice was first evaluated. Next, microarray analysis to investigate the mechanisms behind the protective effect of melatonin was performed. We explored the gene expression profiles mediated by melatonin following radiation. The microarray data were subjected to gene ontology (GO) annotation and ingenuity pathway analysis (IPA). Our data provided fundamental research clues to the radioprotective effects of melatonin and further in-depth study is necessary.

## 2. Results

### 2.1. Melatonin Promoted the Survival of Mice Exposed to Total Body Irradiation (TBI)

**Figure 1:**
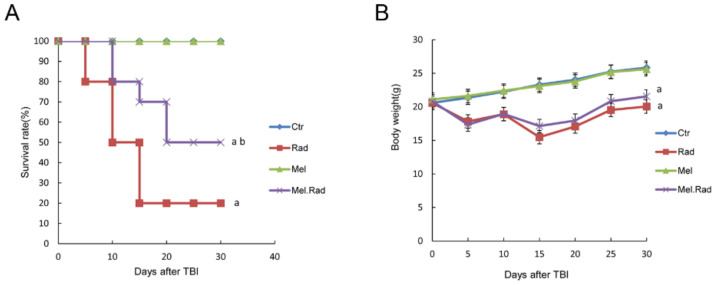
Effects of melatonin on the 30-day survival rate of mice following TBI. Mice were treated with melatonin (10 mg/kg) daily for 5 consecutive days by intraperitoneal injection before exposure to a dose of 7.2 Gy radiation and then examined for 30 days. A group of sham-irradiated control mice was regarded as a control. (**A**) The number of surviving mice was counted and the survival curve was plotted. (**B**) The body weight of surviving mice was detected. Data are represented as mean ± SD, *n* = 10 mice/group. ^a^ *p* < 0.05 vs. Ctr; ^b^ *p* < 0.05 vs. Rad.

The dose range of 7–7.5 Gy radiation was the lethal dose of 50% of many of the experimental mice. To detect the effect of melatonin on fatalness for mice caused by TBI, the 30-day survival rate of mice irradiated with a fatal dose (7.2 Gy) of TBI was first performed (Figure 1A). As demonstrated in Figure 1B, exposure of mice to 7.2 Gy radiation led to a 20% survival rate at 30 days following radiation. However, melatonin administration before radiation significantly increased survival rate to 50%, markedly higher than that of radiation treatment. Meanwhile, the body weight of surviving mice was examined for 30 days following radiation. As shown in Figure 1B, melatonin treatment prior to radiation resulted in an increase in the body weight compared to radiation exposure at 30 days. The data indicate that melatonin administration prior to radiation effectively alleviated TBI-caused fatalness in mice. 

### 2.2. Melatonin Mitigated the Hematopoietic System Damage of Mice Exposed to TBI

**Figure 2:**
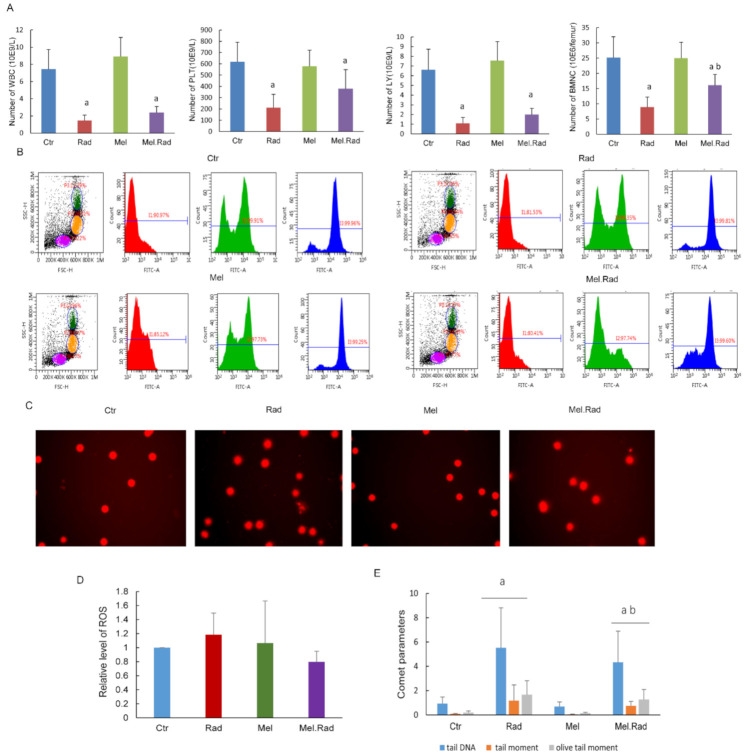
Effects of melatonin on the hematopoietic system of mice following TBI. Mice were treated with melatonin (10 mg/kg) daily for 5 consecutive days by intraperitoneal injection before exposure to a dose of 4 Gy TBI. A group of sham-irradiated control mice was regarded as a control. Whole blood and femurs of mice were harvested at 3 days following radiation. (**A**) The numbers of WBC, PLT, and LY from the whole blood and BMNCs from the femurs were detected. (**B**) Representative ROS images of flow cytometry. (**C**) The ROS levels of the BMNCs were analyzed. (**D**) Representative comet images of DNA damage for lymphocytes. (**E**) The frequency distribution of TDNA, TM, and OTM in lymphocytes was analyzed. Data are represented as mean ± SD, *n* = 6 mice/group. ^a^ *p* < 0.05 vs. Ctr; ^b^ *p* < 0.05 vs. Rad.

The hematopoietic system is susceptible to radiation damage due to its high sensitivity to IR. Exposure of 2–6 Gy radiation was usually used to observe changes to the hematopoietic system in mice. We examined the hematopoietic cell count of the mice exposed to 4 Gy TBI. As shown in Figure 2A, the numbers of WBC, PLT, LY, and BMNCs in the irradiated mice exhibited a clear decrease in comparison to those in the control mice. However, melatonin treatment prior to radiation increased the counts compared to radiation exposure.

Radiation may cause the generation of reactive oxygen species (ROS) in cells. As shown in Figure 2C, there was an increase in ROS level in the bone marrow BMNCs in the Rad mice compared to the Ctr mice. However, melatonin treatment prior to radiation decreased the ROS production. ROS may contribute to extensive oxidative damage to DNA in cells [9]. The effect of melatonin treatment on the DNA damage of lymphocytes from the whole blood was detected by comet assay. As shown in Figure 2E, tail DNA (TDNA), tail moment (TM), and olive tail moment (OTM) from the radiation group obviously increased in comparison to those from the control group. However, there was a significant decrease in the values for the Mel.Rad mice compared to those for the Rad mice. Together, the data suggest that melatonin administration may promote antioxidant activity to alleviate radiation-caused damage on hematopoietic cells. 

### 2.3. Melatonin Alleviated the Damage to the Intestines of Mice Exposed to ABI 

**Figure 3:**
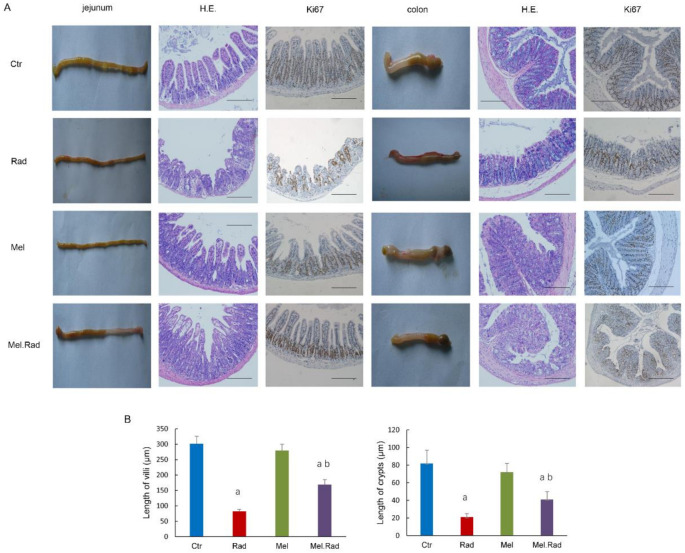
Effects of melatonin on the histomorphology of the intestines of mice following ABI. Mice were treated with melatonin (10 mg/kg) daily for 5 consecutive days by intraperitoneal injection before exposure to a dose of 14 Gy ABI. A group of sham-irradiated control mice was regarded as a control. The jejunum and the colon were dissected for pathological examination at 3 days after radiation. (**A**) Representative pictures of the intestines, H.E. staining, and Ki67-staining. (**B**) The length of villi and crypt of the jejunum. Data are represented as mean ± SD, *n* = 6 mice/group. ^a^ *p* < 0.05 vs. Ctr; ^b^ *p* < 0.05 vs. Rad. Scale bar, 100 μm.

The absorption and barrier functions of the intestines play an important role in protecting intestines from damage. Exposure to radiation may result in discontinuity of the intestinal epithelial barrier, which cannot drive the defensive function of the intestines very well [10]. Exposure to 12–16 Gy abdominal irradiation may be the ideal dose range to determine intestinal recovery. To disclose the effect of melatonin on intestinal injury caused by IR, the small intestines and large intestines were harvested for evaluation by pathological histology after mice were irradiated with 14 Gy ABI. As shown in Figure 3A, the morphology of mucosa in the jejunum and the colon in the irradiated mice was obviously interrupted, with severe loss of villi and crypts, compared to that in the control mice or melatonin-treated mice. By contrast, melatonin treatment prior to radiation resulted in an increase of the length of villi and depth of crypts (Figure 3B). 

Intestinal stems cells (ISCs) are indispensable for maintaining epithelial homeostasis and regeneration of damaged intestines. The brown granules in the villus and the crypt were considered Ki67-positive cells, which indicates that ISCs differentiated into functional cells. As shown in Figure 3A, melatonin administration before radiation resulted in an increasing number of Ki67-positive cells compared to radiation exposure. The data revealed that melatonin promoted the differentiation of ISCs and mitigated the intestinal injury caused by radiation. 

### 2.4. GO Analysis of Gene Expression of the Small Intestine from Mice Exposed to ABI

**Figure 4:**
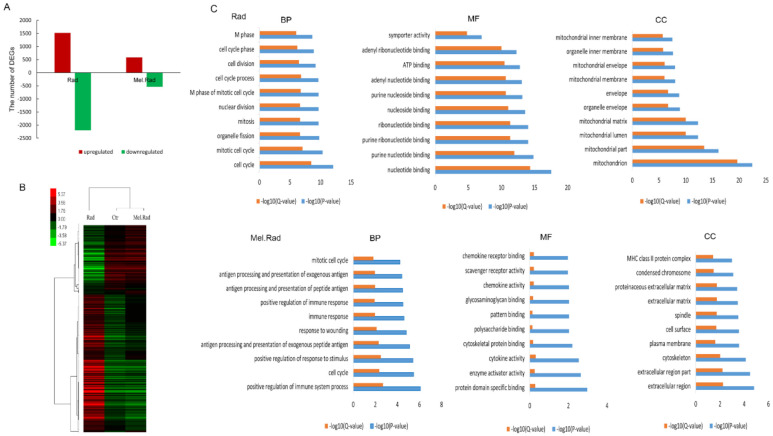
The gene expression profile of the small intestine from mice following ABI. Mice were treated with melatonin (10 mg/kg) daily for 5 consecutive days by intraperitoneal injection before exposure to a dose of 14 Gy ABI. The small intestines were harvested at 3 days after radiation and DNA microarray analysis was performed. (**A**) The number of upregulated DEGs and downregulated DEGs for mice. The red-and-green chart shows the upregulated DEGs (red) and the downregulated DEGs (green). (**B**) Cluster analysis of similarity of genome-wide expression for mice. Every row represents a piece of chip; every line represents a gene probe. The red and green levels of probe signals indicate the level of upregulation (red) or downregulation (green). (**C**) Significantly enriched GO analysis (top10) of the DEGs for mice. The values of −log10 (*p*-value) and −log10 (Q-value) show the relationship between gene expression and the relevant GO category. The *y*-axis represents the GO category and the *x* axis the −log10 (*p*-value) and −log10 (Q-value). *n* = 3 mice/group.

To explore the mechanism of melatonin alleviating IR-caused intestinal injury, we performed DNA microarray analysis to examine the intestinal gene profile in mice following ABI. A total of 3722 differentially expressed genes (DEGs) were found in the Rad mice (1519 upregulated and 2203 downregulated compared to the Ctrl mice), whereas 1122 DEGs were found in the Mel.Rad mice (584 upregulated and 538 downregulated compared to the Ctrl mice) (Figure 4A). Cluster analysis was performed based on 282 gene probes selected by the signal difference of DEGs. As shown in Figure 4B, cluster analysis indicated that genome-wide expression changes in the Rad mice were quite distinct from those in the Ctrl mice or the Mel.Rad mice. The gene expression of Mel.Rad mice was similar to that of the Ctrl mice to some extent. 

To provide further understanding of the data, the DEGs were classified into biological process (BP), molecular function (MF), and cellular component (CC) according to GO annotation analysis. The significantly enriched GO terms for the Rad mice were different from those for the Mel.Rad mice. As shown in Figure 4C, the top10 enriched BP terms in the Rad mice were mainly related to cell cycle, such as cell cycle (BP, GO: 0007049) and mitotic cell cycle (BP, GO: 0000278). For the Mel.Rad mice, the significantly enriched BP terms were mainly involved in the immune process, including positive regulation of immune system process (BP, GO: 0002684), immune response (BP, GO:0006955), and antigen processing and the presentation of exogenous peptide antigen (BP, GO:0002478). Moreover, the significantly enriched MF and CC terms (top10) of the Rad mice were different from those of the Mel.Rad mice (Figure 4C).

### 2.5. IPA of the Small Intestine from Mice Exposed to ABI 

**Figure 5:**
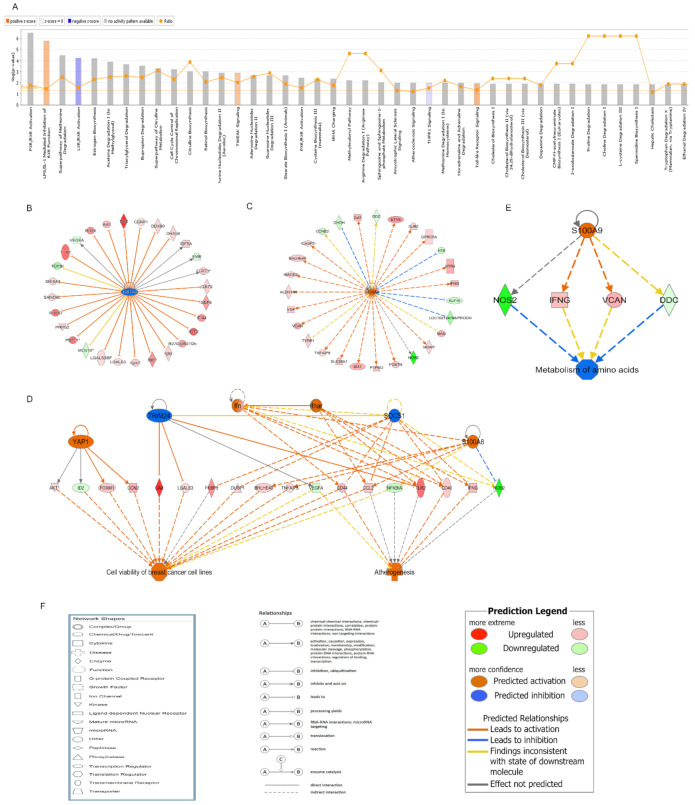
IPA of the small intestines from mice treated with melatonin prior to radiation. (**A**) The diagram of the top 42 canonical pathways the DEGs were involved in was analyzed. The threshold line of canonical pathways represents a *p*-value of 0.05. Bars crossing the threshold line represent significantly changed pathways. The ratio is the number of genes from the data set that map to the pathway divided by the total number of genes that map to the canonical pathway. A Z score >0 represents an activated state, and a Z score <0 an inhibited state. (**B**) Upstream regulatory network of TRIM24 and its targets. (**C**) Upstream regulatory network of S100A9 and its targets. (**D**) Regulator effects network that TRIM24 was involved in. (**E**) Regulator effects network that S100A9 was involved in. (**F**) The representative meaning of the symbols in the figures. Genes or gene products are expressed as nodes, and the biological co-relationship between two nodes is indicated by the connecting lines. Nodes are represented using diverse shapes that express the functional class of the gene product. The degree of the node color indicates the level of upregulation (red) or downregulation (green). *n* = 3 mice/group.

IPA is a robust and expertly curated database that can help to understand the characters of various molecules such as gene, protein, chemical substance, and drug and interaction network among the molecules [11]. The data from Mel.Rad mice were further analyzed by IPA to group the identified genes and locate the canonical pathways that the genes were involved in. The diagram of the top 42 pathways of melatonin treatment prior to radiation is shown in Figure 5A. LPS/IL-1 mediated inhibition of RXR Function, TWEAK signaling, and Toll-like receptor signaling were the most activated canonical pathways, and the LXR/RXR activation and TNFR1 signaling were the most inhibited canonical pathways.

Upstream regulators based on all DEGs in the IPA database overlaid with microarray data were analyzed. It was found that 56 upstream regulators were inhibited and 71 activated. Tripartite motif-containing 24 (TRIM24) was forecasted to be the most significantly inhibited (Z = −4.082) and S100 calcium binding protein A9 (S100A9) activated (Z = 3.53). The network of TRIM24 and S100A9 transcription regulators and their downstream targets was built. TGFBI and MOV10 were predicted to be activated; EIF5A, EMB, FLRT3, and VEGFA affected; and 22 other downstream targets inhibited by TRIM24 (Figure 5B). MAG, TYRP1, DDC, and CCNE2 were predicted to be inhibited; NOS2 affected; and 22 other downstream targets activated by S100A9 (Figure 5C). 

The regulator effects network demonstrated the interaction between the DEGs and regulators in the data sets and their function. The regulatory network TRIM24 was involved in showed that TRIM24, YAP1, Ifn, Ifnar, SOCS1, and S100A8 together activated atherogenesis and cell viability of breast cancer cell lines through 18 genes, including AKT1, ID2, etc. (Figure 5D). The regulatory network S100A9 was involved in showed that S100A9 inhibited the metabolism of amino acids through DDC, IFNG, NOS2, and VCAN (Figure 5E).

### 2.6. qRT-PCR Validation of the Candidate DEGs Involved in the Regulatory Network of TRIM24 and S100A9 

**Figure 6:**
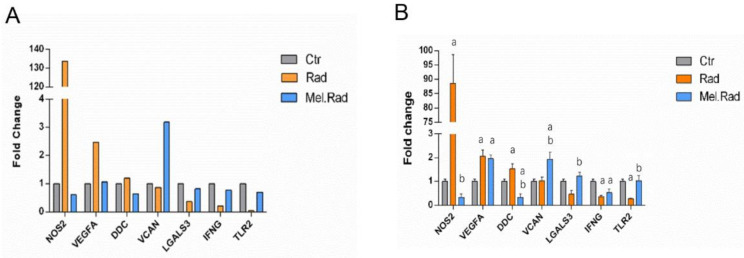
qRT-PCR validation of seven candidate DEGs. (**A**) The expression levels of the target genes of TRIM24 and S100A9 in the microarray data. (**B**) The expression levels of the target genes of TRIM24 and S100A9 validated in the qRT-PCR. Data are represented as mean ± SD, *n* = 3 mice/group. ^a^ *p* < 0.05 vs. Ctr; ^b^ *p* < 0.05 vs. Rad. NOS2, nitric oxide synthase 2, inducible; VEGFA, vascular endothelial growth factor A; DDC, dopa decarboxylase; VCAN, versican; LGALS3, lectin, galactose binding, soluble 3; IFNG, interferon gamma; TLR2, toll-like receptor 2.

Seven candidate DEGs based on the downstream targets TRIM24 and S100A9 involved in were discerned to perform qRT-PCR, including LGALS3, TLR2, VEGFA, DDC, IFNG, NOS2, and VCAN. In the microarray data, the expression levels of the genes NOS2, VEGFA, and DDC were significantly upregulated, and VCAN, LGALS3, IFNG, and TLR2 downregulated in the Rad mice compared to the Ctrl mice. The levels of VCAN, LGALS3, IFNG, and TLR2 increased, and NOS2, VEGFA, and DDC decreased in the Mel.Rad mice compared to the Rad mice (Figure 6A). As shown in the qRT-PCR validation result, most of the expression levels were basically consistent with those of the microarray data (Figure 6B).

### 2.7. Validation of the Effect of Melatonin Treatment on the Immunity 

**Figure 7:**
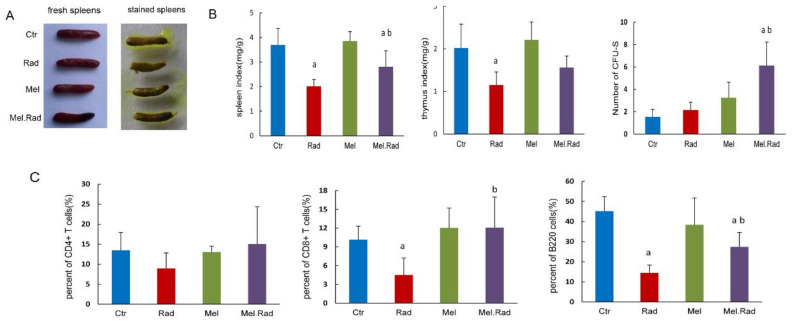
Effects of melatonin treatment on the immune system of mice following ABI. Mice were treated with melatonin (10 mg/kg) daily for 5 consecutive days by intraperitoneal injection before exposure to a dose of 14 Gy ABI. A group of sham-irradiated control mice was regarded as a control. Spleen, thymus, and whole blood of mice were harvested at 3 days following radiation. (**A**) Representative images of the spleens. (**B**) The spleen/thymus coefficient and CFU-S per spleen were counted. (**C**) CD4^+^ T cells, CD8^+^ T cells, and B cells in blood were analyzed. Data are represented as mean ± SD, *n* = 6 mice/group. ^a^ *p* < 0.05 vs. Ctr; ^b^ *p* < 0.05 vs. Rad.

The bioinformatic analysis indicated that the radioprotective effects of melatonin on radiation damage were associated with activation of the immune response. To determine whether melatonin can promote the immunity of the body following radiation, we examined the effect of melatonin treatment on the immune organs and immune cells. It was found that the spleen/thymus coefficient of the Rad mice was lower than that of the Ctrl mice. However, melatonin treatment increased the spleen/thymus coefficient more than radiation exposure (Figure 7B). Similarly, melatonin treatment pre-radiation demonstrated a significant increase in colony-forming unit-spleen (CFU-S) compared to radiation exposure. As shown in Figure 7C, the analysis of lymphocyte phenotypes indicated that melatonin administration before radiation resulted in a significant increase in the number of CD8^+^ T cells and B cells compared to radiation exposure. The data suggest that melatonin administration prior to radiation stimulates the body’s immunity.

## 3. Discussion

In the study, the possible effects of melatonin on radiation damage in mice were first investigated. Survival rate is regarded as an important parameter indicating radiation effects. We found that melatonin promoted the survival rate of mice irradiated with a fatal dose of TBI, suggesting the radioprotective effect of melatonin on the body. 

The hematopoietic system is extremely sensitive to radiation during radiotherapy, which may result in impaired hematopoietic function [12]. In the study, melatonin treatment prior to radiation increased the counts of whole blood cells and bone marrow nucleated cells. DNA is the primary target for the lethal effects of radiation. ROS induced by radiation may destroy the structure and function of DNA, resulting in oxidative damage to cells [13]. Because of its small size and high lipophilicity, melatonin can easily enter cellular membranes, reaching its highest concentration in the nucleus, and thus protecting the DNA of the nucleus from oxidative injury caused by IR [14]. We found that melatonin decreased the generation of ROS in bone marrow and alleviated radiation-caused DNA damage of lymphocytes in whole blood. The data suggest that melatonin may alleviate radiation-caused damage on hematopoietic cells by promoting antioxidant activity. It has been well established that mice irradiated with fatal doses of radiation that survive depend on the restoration of the hematopoietic system [15]. Therefore, the survival rate and the body weight of the melatonin-treated mice prior to radiation was improved in the study. 

To disclose the effect of melatonin on radiation-caused intestinal injury, the intestines were evaluated by pathological histology. The results indicate that the impaired intestinal villus and severe loss of crypts caused by radiation were recovered by melatonin treatment. Thus, melatonin facilitated the recovery of absorption and barrier functions of the intestines and alleviated the intestinal injury caused by radiation.

In exploring the mechanisms of the radioprotective function of melatonin on intestinal injury, melatonin treatment before radiation resulted in significant changes in DNA expression patterns of the small intestines. GO analysis clearly showed that the significantly enriched biological process mainly included the immune process in the mice treated with melatonin. Many studies have shown that melatonin has a positive effect on immunity [16]. In the IPA analysis of canonical pathways, we found that the most activated pathways affected by melatonin treatment prior to radiation included LPS/IL-1-mediated inhibition of RXR function and Toll-like receptor signaling. Lipopolysaccharide (LPS), a principal component of the outer membrane of Gram-negative bacteria, strongly stimulates host innate immune response [17]. Toll-like receptors (TLRs) are usually expressed in innate immune cells and non-immune cells such as epithelial cells and fibroblasts, and react to the membrane constituents of Gram-positive or Gram-negative bacteria [18]. LPS recognition as a constituent of bacterial outer membrane by TLRs evokes rapid activation of innate immunity. The two activated pathways were involved in innate immune responses, suggesting that the activation of innate immunity plays an important role in the protective effect of melatonin against IR. 

To determine the effect of melatonin on innate immunity, we examined the change in immune organs and immune cells following melatonin treatment. The spleen is the largest lymphoid organ in the human body and the colony-forming ability of the spleen was used to evaluate the immune function following exposure to high doses of IR [15]. We found that melatonin administration resulted in an increase in the CFU-S and spleen/thymus coefficient. A similar effect of melatonin treatment on CD4^+^ T-cell, CD8^+^ T-cell, and B-cell recovery in blood was also observed in the irradiated mice. The data indicated that melatonin treatment motivated the recovery of immunity following IR and immune regulation was related to the protective effect of melatonin on intestinal injury. It was reported that a significantly enriched biological process was involved in innate and adaptive immunity in male mice treated with sex-matched fecal microbiota transplantation to alleviate intestinal injury [19], which was consistent with our results. 

In further analysis of the upstream regulatory network and regulator effects network affected by melatonin treatment before radiation, TRIM24 was forecasted to be the most significantly inhibited upstream regulator. TRIM24 is a member of the tripartite motif family, which plays an important role in regulation of innate immunity, autophagy, and carcinogenesis [20]. TRIM24 was reported to regulate the activity of the retinoic acid receptor and the tumor suppressor p53 [21]. Diseases such as differentiated thyroid carcinoma, hepatocellular carcinomas, and cerebellar agenesis were associated with TRIM24. In the study, the regulatory network of TRIM24 activated atherogenesis and cell viability in breast cancer cell lines. In addition, we found that S100A9 was forecasted to be the most significantly activated upstream regulator. S100A9 is a calcium- and zinc-binding protein. Usually, S100A9 and S100A8 are secreted in a heterodimeric form by activating immunocytes such as monocytes, granulocytes, and neutrophils [22], which are involved in the regulation of inflammatory processes and immune response [23]. In the study, the regulatory network of S100A9 inhibited the metabolism of amino acids. 

In the qRT-PCR validation of the candidate DEGs involved in the regulatory network of TRIM24 and S100A9, there was an obvious increase and decrease in the mRNA levels of TLR2 and NOS2, respectively, in the small intestines of mice treated with melatonin. TLR2 is expressed on many cells such as macrophages, dendritic cells, and monocytes that are responsive to peptides from bacteria. TLR2 participated in the two signaling pathways, Toll-like receptor signaling and LPS-mediated signaling [24]. The increased TLR2 level in the Mel.Rad mice denoted that immune response was activated following melatonin treatment. NOS2, a nitric oxide synthase, resulted in inflammation by increasing the synthesis of proinflammatory mediators such as IL6 and IL8 [25]. The decreased NOS2 level in the Mel.Rad mice suggested that inflammation was inhibited by melatonin’s immune defense.

We preliminarily explored the mechanisms of the radioprotective effect of melatonin on intestinal injury, which were associated with activation of the immune response. However, the relationship between the two activated pathways involved in immunity, LPS/IL-1-mediated inhibition of RXR function, and Toll-like receptor signaling was not investigated in this study. So far, the roles of TRIM24 and S100A9 in the regulatory network associated with melatonin treatment following radiation have not been reported. How TRIM24 and S100A9 participate in the beneficial effect of melatonin are still not very clear. It is required to conduct in-depth experiments to facilitate the understanding of melatonin as a radioprotector for radiation enteropathy. 

Studies on healthy male volunteers by oral administration of melatonin in doses of 1–300 mg [26] or 1 g of melatonin daily for 30 days [27] reported no evidence of toxicity. In addition, some randomized clinical trials indicated that administration of melatonin (20 mg/day) improved the quality of life of cancer patients and thus demonstrated a therapeutic role of melatonin [28,29]. Melatonin administration, either alone or combined with radiotherapy, resulted in a favorable outcome. Melatonin has the potential as a radioprotector in the radiotherapy for tumor patients.

In conclusion, the data provided a scene of gene expression of melatonin treatment before radiation and fundamental research clues to the protective effect of melatonin. Melatonin might promote immune response and thus protect the intestines from radiation damage. However, the associated mechanisms of activation of the immunity have not yet been elucidated thoroughly. It is necessary to conduct further intensive experiments to explore the underlying mechanisms of melatonin’s immune regulation on intestinal injury.

## 4. Materials and Methods

### 4.1. Mice

Male C57BL/6J mice were obtained from Vital River (Beijing, China). Mice 6–8 weeks of age and with 18 ± 2 g of body weight were used. The mice were raised under laboratory conditions of 23 ± 2 °C temperature, 50 ± 5% humidity, and light/dark 10/14 h. The mice were kept in the experimental animal center at the Institute of Radiation Medicine of the Chinese Academy of Medical Science. The mice were humanely euthanized through 100% CO_2_ inhalation, in accordance with the principles guidelines of the Institutional Animal Care and Ethics Committee guidelines.

### 4.2. Radiation Exposure in Mice 

The mice were anaesthetized with chloral hydrate solution (0.3 g/kg body weight) by intraperitoneal injection. The mice were restrained in a perforated wooden container and radiation was delivered to the abdominal area with the other parts of the body covered by lead plate. The mice were irradiated with a ^137^Cesium γ irradiator (Atomic Energy of Canada Inc., Chalk River, ON, Canada) at a rate of 0.883 Gy/min. 

### 4.3. Administration of Melatonin

Melatonin (*N*-acetyl-5-methoxytryptamine) was purchased from Sigma-Aldrich (St. Louis, MO, USA). The melatonin was dissolved in 50 μL absolute ethanol and diluted with isotonic NaCl solution to a final ethanol concentration of 10%. The mice were treated with 10 mg/kg body weight of melatonin by intraperitoneal injection. The dose of melatonin was selected based upon our previous study [30].

### 4.4. Experimental Design

The animals were randomly split up into four groups: (i) Ctrl group, mice received no treatment. (ii) Rad group, mice were irradiated with a certain dose of gamma irradiation once. (iii) Mel group, mice were administered with melatonin by intraperitoneal injection. (iv) Mel.Rad group, mice were administered melatonin by intraperitoneal injection. One hour following the last administration, the mice were irradiated with a certain dose of gamma irradiation once. Throughout the experiment, melatonin was administered between 4 p.m. and 5 p.m. Melatonin is regarded as being at its lowest natural concentration in the blood during this time period.

### 4.5. Analysis of 30-Day Survival Rate

The mice were treated based on the grouping method. The mice were observed for up to 30 days following a fatal dose of 7.2 Gy TBI. The number of surviving animals and the body weight of the surviving animals was examined. The survival curve of the animals was drawn by the Kaplan–Meier method.

### 4.6. Pathological Analysis

The intestines of the mice were dissected and immersed in 4% neutral buffered formalin for 24 h. The paraffin-embedded sections were sliced and dyed with hematoxylin and eosin staining. The slides were observed with a microscope (BX51, Olympus, Shinjuku, Tokyo, Japan).

### 4.7. Immunohistochemical Analysis

Paraffin-embedded slides were dewaxed and rehydrated with xylene and ethanol, respectively, and put in 3% hydrogen peroxide solution for 10 min to block endogenous peroxidase. The slides were immersed in Tris/EDTA (pH 9.0) and incubated for 15 min at 98 °C to retrieve antigens. The slides were incubated with serum for 30 min at room temperature to block nonspecific antigen binding sites. Mouse monoclonal anti-Ki67 antibody was diluted (Abcam Biotechnology, Cambridge, UK, No. ab8191, 1:1000 dilution), added to the slides, and incubated overnight at 4 °C. Secondary antibody HRP goat anti-mouse (IgG) antibody (Abcam Biotechnology, Cambridge, UK, No. ab205719) was added and incubated at 37 °C for 2 h.

### 4.8. Peripheral Blood Cell and Bone-Marrow Cell Counts

A volume of 0.2 mL of whole blood was drawn from the orbital sinuses of mice by a micropipette coated with an anticoagulant. The cells, including white blood cells (WBC), platelets (PLT), and lymphocytes (LY), were counted with the hemocytometer (Sysmex pocH-100i; Sysmex Corporation, Kobe, Japan). Bone marrow nucleated cells (BMNCs) were collected from the femurs as per previous literature and counted [31].

### 4.9. Reactive Oxygen Species (ROS) Level Assay

BMNCs were collected from the femurs. The levels of ROS were detected by ROS assay kit 520 nm (Invitrogen, Carlsbad, CA, USA) using a flow cytometer. A 1 × ROS assay stain solution (100 μL) was added to each sample and incubated for 60 min at 37 °C. Then the solution was washed twice with PBS solution and re-suspended in 500 μL PBS solution. A minimum of 10,000 cells for each sample were collected and analyzed using FlowJo 7.6.1 software (Tree-Star, Ashland, OR, USA).

### 4.10. Analysis of DNA Damage in Lymphocytes and Lymphocyte Phenotype

A volume of 0.2 mL of whole blood was drawn from the orbital sinuses of mice. Lymphocytes were isolated with the lymphocyte separation medium and a comet assay was carried out in alkaline conditions as described previously [32]. The slides were observed using an ECLIPSE 90i fluorescence microscope (Nikon, Tokyo, Japan). A total of 100 comet images were collected for each sample with a digital imaging system (Cherry Hill, NJ, USA) and analyzed using the Comet Assay Software Project (CASP, Wroclaw, Poland) [33]. The proportion of DNA in the tail DNA (TDNA), tail moment (TM), and olive tail moment (OTM) were calculated to record the degree of DNA damage. A lymphocyte phenotype assay was carried out by flow cytometry as described previously [34].

### 4.11. cDNA Microarray Analysis

The small intestines of mice were harvested and immediately immersed in RNA stabilization reagent (Qiegen, Shanghai, China). Total RNA was drawn with TRIzol reagent (Invitrogen, Carlsbad, CA, USA) and reverse transcribed to complementary DNA using the Quantscript RT kit (Tiangen Biotech, Beijing, China). The aminoallyl-RNA probes were labelled with Cy5 hybridized for 16 h at 50 °C to a mouse oligo microarray (Mouse Whole Genome OneArray MOA2.1; Phalanx Biotech Group, Hsinchu, Taiwan). All arrays were scanned using Agilent Microarray Scanner G2505C at a 10 μm resolution. Raw data were obtained from the scanned images and analyzed with Rosetta Resolver 7.2 software (Rosetta Biosoftware Technologies, San Diego, CA, USA). The PLIER default protocol was used to normalize the data.

### 4.12. GO Annotation and IPA Analysis

DEGs with at least 2-fold change in expression level with a *p*-value (differentially expressed) less than 0.05 were regarded to be significantly upregulated or downregulated. To gain insight into the levels of DEGs identified by cDNA microarray, GO annotation analysis was performed, including CC, MF, and BP. The GO terms were considered to be significantly enriched when the *p*-value (differentially expressed) was less than 0.05. Microarray data were analyzed by IPA (Ingenuity Systems, Redwood City, CA, USA) to ascertain the canonical pathways, upstream regulation, and regulator effect based on the whole DEGs involved in and previous literature.

### 4.13. qRT-PCR

Total RNA was isolated from the small intestines with TRIzol reagent (Invitrogen, Carlsbad, CA, USA) and reverse transcribed to complementary DNA was synthesized with an RNA PCR kit. The qPCR experiment was conducted using a SYBR Green kit (Takara Bio Inc., Dalian, China). The primer sequences were as follows: LGALS3: 5′-AGACAGCTTTTCGCTTAACGA-3′ (forward) and 5′-GGGTAGGCACTAGGAGGAGC-3′ (reverse), TLR2: 5′-GCAAACGCTGTTCTGCTCAG-3′ (forward) and 5′-AGGCGTCTCCCTCTATTGTATT-3′ (reverse), VEGFA: 5′-GAGGTCAAGGCTTTTGAAGGC-3′ (forward) and 5′-CTGTCCTGGTATTGAGGGTGG-3′ (reverse), DDC: 5′-TAGCTGACTATCTGGATGGCAT-3′ (forward) and 5′-GTCCTCGTATGTTTCTGGCTC-3′ (reverse), IFNG: 5′-TCCTCGCCAGACTCGTTTTC-3′ (forward) and 5′-GTCTTGGGTCATTGCTGGAAG-3′ (reverse), NOS2: 5′-GTTCTCAGCCCAACAATACAAGA-3′ (forward) and 5′-GTGGACGGGTCGATGTCAC-3′(reverse), VCAN: 5′-TTTTACCCGAGTTACCAGACTCA-3′ (forward) and 5′-GGAGTAGTTGTTACATCCGTTGC-3′ (reverse), and GAPDH: 5′-AGGTCGGTGTGAACGGATTTG-3′ (forward) and 5′-TGTAGACCATGTAGTTGAGGTCA-3′ (reverse). The primers were synthesized by Sangon Biotech (Shanghai, China).

### 4.14. Calculation of CFU-S and Spleen/Thymus Coefficient

The spleens and thymuses were dissected out from the abdominal cavity after the mice were euthanized. The spleens were stained using picric acid for 24 h and observed under a macroscope to count CFU-S. One nodule on the spleen surface that was visible to the naked eye was considered one CFU-S [35]. The spleen/thymus coefficient was obtained by dividing the weight of a single spleen/thymus (mg) by the body weight (g).

### 4.15. Statistical Analysis

Each experiment was performed at least three times. Data are shown as mean ± SD. The 30-day survival rate was compared using the Kaplan–Meier method with a log-rank test. Statistical differences between groups were analyzed by one-way ANOVA followed by the Bonferroni post hoc test. A *p*-value less than 0.05 was considered to be statistically significant.

## Data Availability

Not applicable.
